# A decade of innovation to deepen the understanding of infectious diseases of poverty and foster their control and elimination

**DOI:** 10.1186/s40249-022-01037-0

**Published:** 2022-10-24

**Authors:** Dirk Engels, Sheng-lan Tang, Colin D. Butler, Ayoade M. J. Oduola, Tania C. de Araujo-Jorge, George F. Gao, Jürg Utzinger, Xiao-Nong Zhou

**Affiliations:** 1 World Health Organization (Retired), Geneva, Switzerland; 2 Duke University, Durham, USA; 3 Australia National University, Canberra, Australia; 4 University of Ibadan Research Foundation, University of Ibadan, Ibadan, Nigeria; 5 Institute Oswaldo Cruz, Oswaldo Cruz Foundation, Rio de Janeiro, Brazil; 6 Chinese Academy of Sciences, Beijing, People’s Republic of China; 7 Swiss Tropical and Public Health Institute, Allschwil, Switzerland; 8 University of Basel, Basel, Switzerland; 9 National Institute of Parasitic Diseases at Chinese Center for Disease Control and Prevention, Chinese Center for Tropical Diseases Research, Shanghai, People’s Republic of China; 10 One Health Center, Shanghai Jiao Tong University-The University of Edinburgh, Shanghai, People’s Republic of China

Exactly 10 years ago, on 25 October 2012, a group of scientists, policy-makers, and practitioners undertook an ambitious project to launch a new open-access journal, named *Infectious Diseases of Poverty* [1]. The idea had originated from a think-tank put forth by the Special Programme for Research and Training in Tropical Diseases (TDR), an organisation co-sponsored by the United Nations Children’s Fund (UNICEF), the United Nations Development Programme (UNDP), the World Bank, and the World Health Organization (WHO). Indeed, this think-tank published a comprehensive *Global Report for Research on Infectious Diseases of Poverty* [2] that guided the scope and remit of *Infectious Diseases of Poverty*.

The aim of the new journal was to publish a blend of original research articles and more empirical work, the latter embracing different types of articles, including scoping reviews, case studies, opinion pieces, and policy briefs. Its focus was to be on infectious diseases of poverty, coinciding with the launch of WHO’s first roadmap to overcome the global impact of neglected tropical diseases (NTDs) earlier that year [3], and underpinning the Millennium Development Goals (MDGs), particularly to eradicate extreme poverty and hunger (MDG 1) and to combat HIV/AIDS, malaria, and other diseases (MDG 6). The journal also aimed to build on the “One Health, One World” approach, and to set the stage for a new strategic direction in research and innovation on infectious diseases of poverty by bolstering trans-disciplinary work [1, 4–6].

The event to celebrate the 10th anniversary of continuous publication of the journal, *Infectious Diseases of Poverty,* provides a juncture to review the relationship between the scope of publications and the journal’s impact. During the last decade, the journal has endorsed and promoted the One Health discipline, which has evolved from concept to practices [7], and from the first *One World, One Health* Conference formulating the Manhattan principles in 2004, to the action plan that has been formulated by four international organizations, namely the Food and Agriculture Organization of the United Nations (FAO), the World Organisation for Animal Health (WOAH/OIE), the United Nations Environmental Programme (UNEP), and WHO. Over the years, the number of publications and their citations of One Health research has grown considerably [8]. In parallel, the global pursuit of the attainment of the MDGs has evolved into the broader concept of the Sustainable Development Goals (SDGs), while poverty alleviation programmes have brought significant changes in human health and wellbeing, particularly to deprived populations in remote areas.

The impact factor of *Infectious Disease of Poverty* has been climbing up as well, exceeding 10 in 2021 with an *H*-index of 47, according to the Web of Sciences (accessed on 6 October 2022). Importantly, the journal continues to engage participants from the low- and middle-income countries (LMICs) in the leadership, management and dissemination of its outputs. These developments are complemented by a series of remarkable milestones (Fig. 1). Hence, in order to better understand the real impact of the journal, we carefully examined whether the journal’s original goals have been achieved, such as the recognition and reinforcement of the nexus of research outputs on health in the field of poverty alleviation, food security, sustainable agriculture, social justice, economic prosperity, and environmental protection [9].

**Fig. 1:**
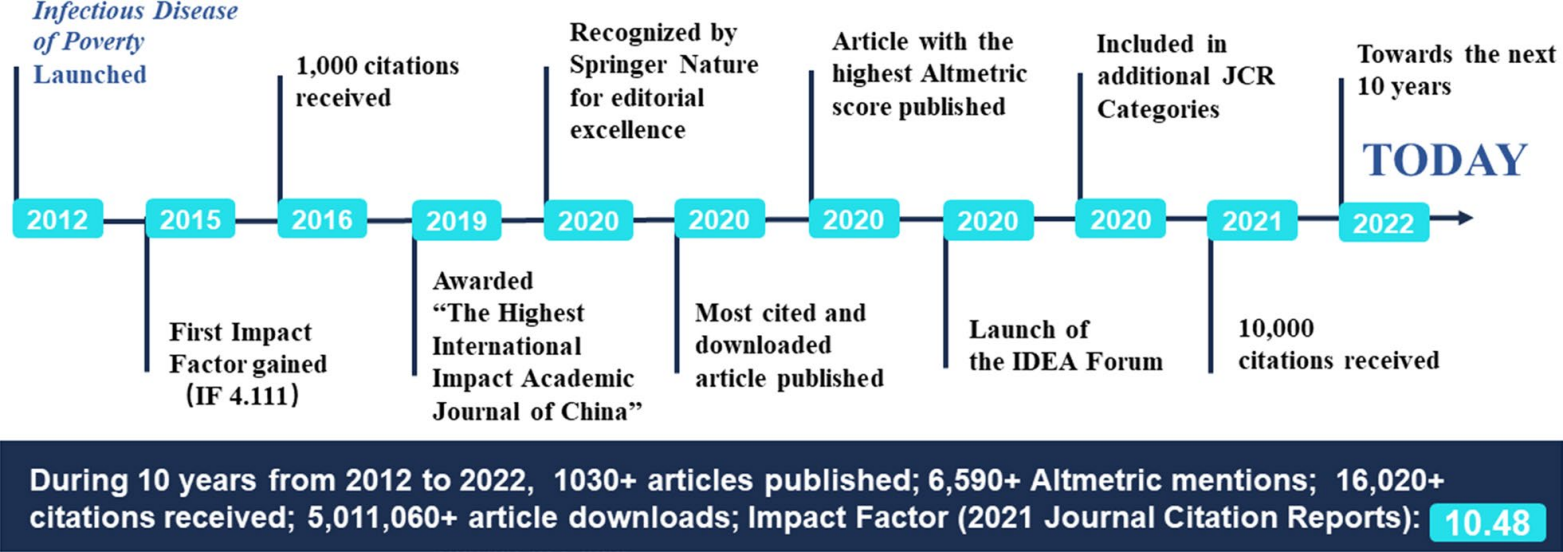
Milestones of *Infectious Diseases of Poverty* in the first decade (journal launched on 25 October 2012; analysis pursued in September 2022)

Firstly, the journal has published a large amount of original and high-quality research in the field of infectious diseases of poverty. A particular aspect of the journal that merits highlighting is the publication of thematic series and article collections. Thus far, a total of 34 thematic series have been published with 441 articles, meaning an average of 3 thematic series and more than 40 articles per year. An example is the collection "Health systems research for infectious diseases of poverty" that was introduced in the inaugural issue of *Infectious Diseases of Poverty,* and has reviewed in considerable depth the role of health systems in combating infectious diseases of poverty. The thematic issue “Ebola outbreaks and community-based surveillance response systems”, published in 2016, has accumulated knowledge on the human-animal interplay and has identified information gaps, community-based control strategies, and the importance of translational research. The thematic series “Schistosomiasis research: providing the tools needed for elimination” was also launched in 2016 and aimed to validate new techniques in support of WHO’s goal of worldwide elimination of the disease. The topical collection “Urban health and prevention and control of vector-borne diseases” was launched in 2018 in collaboration with TDR and has identified research priorities on interventions for vector-borne diseases in the urban environment. In the initial stage of the COVID-19 outbreak, the journal launched a thematic collection “Transmission patterns and control of COVID-19 epidemic”, that provided timely and valuable scientific evidence to help containing the pandemic. Articles in this collection have been downloaded more than one million times, and cited nearly 3000 times.

Secondly, the journal has been building an academic community working on infectious diseases of poverty, insisting on the value of integrating transdisciplinarity and systems thinking [10, 11]. On specific world disease days, the journal published a corresponding paper and promoted the creation of an academic atmosphere to draw the attention from international peers. For instance, an editorial published on World Rabies Day, on 26 September 2013, proposed approaches to move from the biological understanding to the science of rabies elimination [12]. On the World Neglected Tropical Diseases Day, on 30 January 2021, the journal published a paper which analysed the relationship between the new COVID-19 poor' and NTDs resurgence and called for discussion of this issue in the World Health Assembly [13].

To meet the readers’ needs, more distinctive article types, such as Scoping Review, Policy Brief, and Study Protocol, have been introduced. As such, the journal has fostered young scientists to accumulate the scientific evidence for policy-making and to promote implementation of policies to combat infectious diseases of poverty in their own settings. To cite an example, Dr. Ernest Tambo from Cameroon has progressed with the journal, starting with submitting a doctoral protocol and further with contributing to the journal by organizing thematic series, identifying research gaps, and making recommendations for the prevention, control, and elimination of infectious disease of poverty in African settings. Similarly, the journal has nurtured about 5000 young scientists on competencies to design, report, and communicate research findings in an accessible and concise manner, through scientific writing training courses and IDEA (idea, design, editing, article) fora.

Thirdly, the journal has made efforts to disseminate seminal research findings by 18 international channels, such as e-mail campaigns, editor picks, landing pages, cross-journal promotion, among others. This has substantially improved the visibility of the journal, the articles, and the authors of the respective papers. Multidisciplinary topics and promotional efforts have made the journal a reputable and valued academic platform for scientists, policy-makers, professionals, and students in the field of infectious diseases and One Health. As of September 2022, the journal has published 1060 papers by 5298 authors from 108 countries, that were downloaded over 4.7 million times. Such a huge amount of dissemination has facilitated collaboration between scientists worldwide. Yet, an analysis of authors’ cross-country collaborations has shown that the People’s Republic of China, the United States of America, the United Kingdom, Switzerland, and European countries still constitute most of the research-centres of collaboration in the field of infectious diseases of poverty, and that much more remains to be done to foster key competencies to conduct programmes from tropical and sub-tropical regions (Fig. 2). As such, an increase in publications coming from Central and South America is still a challenge for the journal compared with the number of publications from other regions.

**Fig. 2:**
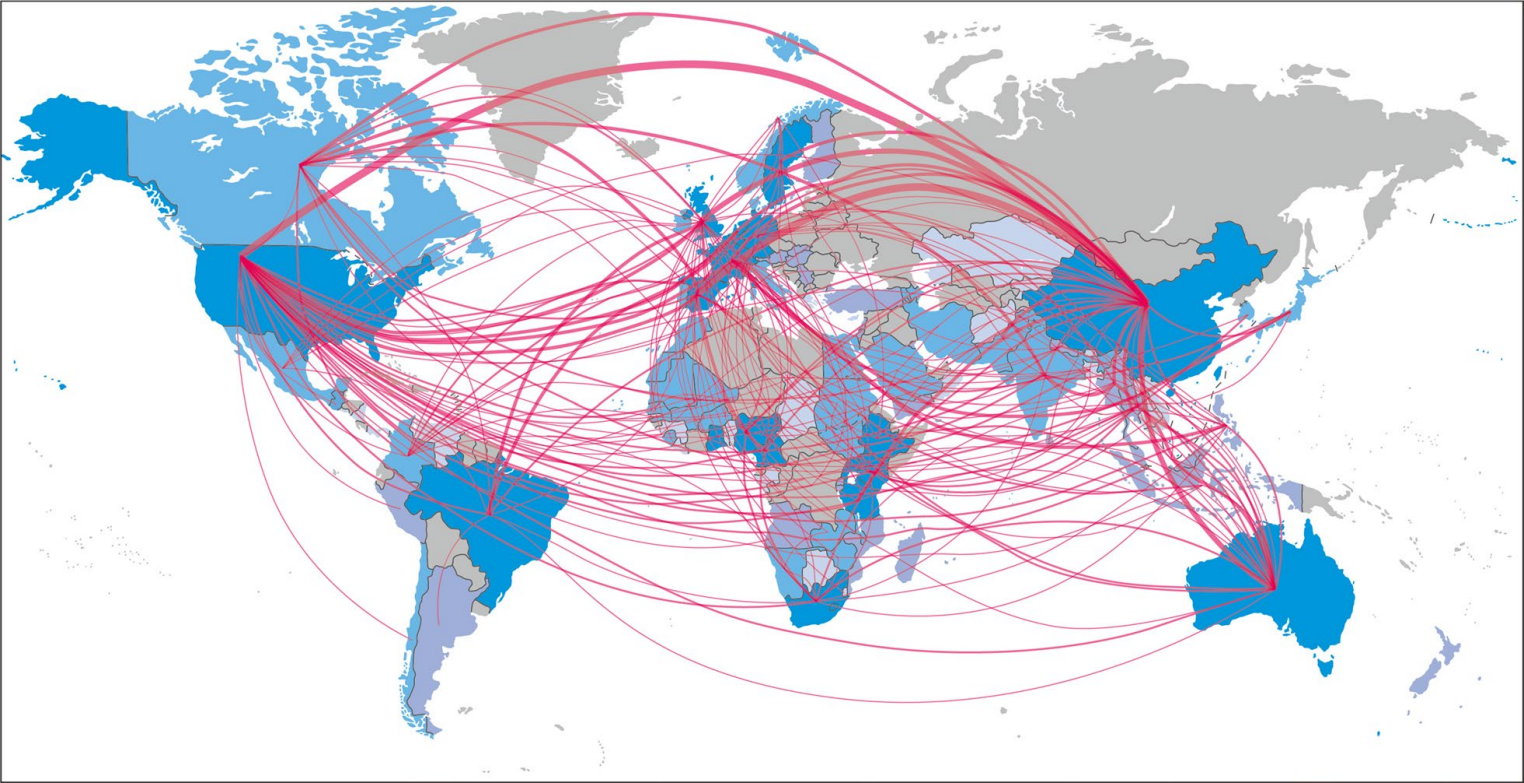
The pathway of the collaborators around the world based on all publications in *Infectious Diseases of Poverty* from 2012 to 2022, showing the research-centres of those collaborators located in the People’s Republic of China, United States of America, United Kingdom, Switzerland, and elsewhere

As programmes to prevent, control, and eliminate infectious diseases of poverty have considerably been hampered by the COVID-19 pandemic with an estimated delay in the realization of the SDGs by 2030 [14], the journal will intensify its efforts to explore new avenues in research to better understand the relationship between infectious diseases and poverty, and construct a rationale to boost plans to control them. In the near future, the scope of the journal will continue to focus on One Health research, with the following priorities: (i) control of ancient NTDs, study of emerging and re-emerging infectious diseases, and better understand the interface between humans, animals, and the environment; (ii) research on new tools and strategies for the diagnosis, control and elimination of communicable and non-communicable diseases, such as the application of sequencing technologies to detect and predict new emerging pathogens; (iii) identification of research gaps in currently known scientific evidence, and connection with policy formulation and implementation; (iv) boosting more programmes on vaccine-preventable diseases in LMICs; and (v) standardization of publications in Scoping Review and Policy Brief that are expected to bridge and help communications between researchers and policy-makers, and to promote the translation of research findings to improve health and well-being of people, animals, and the environment, thus embracing planetary health [15]. In the long-term, the ambition of the journal is to foster the capacity to combat infectious diseases among young scientists whose countries or communities are affected by these diseases, especially researchers from low-income countries. The latter can be routinely granted for waiving of the article-process charge (APC). For other countries, APC waivers or discounts are granted on a case-by-case basis to authors with insufficient funds. The journal will strive to accompany authors in their high-level studies from design to report, and increase the number of young editorial board members to make youth—blended with more seasoned editors—a driving force to further build and entertain an academic community in the field of infectious diseases of poverty.

## Data Availability

All data used in this paper has been presented.

## Abbreviations

APC: Article-Process Charge
FAO: Food and Agriculture Organization of the United Nations
LMICs: Low- and Middle-Income Countries
MDGs: Millennium Development Goals
NTDs: Neglected Tropical Diseases
SDGs: Sustainable Development Goals
TDR: Special Programme for Research and Training in Tropical Diseases
UNDP: United Nations Development Programme
UNEP: United Nations Environmental Programme
UNICEF: United Nations Children’s Fund
WHO: World Health Organization
WOAH/OIE: World Organisation for Animal Health
